# Improving medication adherence in diabetes type 2 patients through Real Time Medication Monitoring: a Randomised Controlled Trial to evaluate the effect of monitoring patients' medication use combined with short message service (SMS) reminders

**DOI:** 10.1186/1472-6963-11-5

**Published:** 2011-01-10

**Authors:** Marcia Vervloet, Liset van Dijk, Jacqueline Santen-Reestman, Bas van Vlijmen, Marcel L Bouvy, Dinny H de Bakker

**Affiliations:** 1 NIVEL, Netherlands institute for health services research, P.O. Box 1568, 3500 BN Utrecht, The Netherlands; 2 Mediq Apotheken, P.O. Box 2450, 3500 GL Utrecht, The Netherlands; 3 Utrecht Institute of Pharmaceutical Sciences, Division of Pharmacoepidemiology and Clinical Pharmacology, Utrecht University, P.O. Box 80082, 3508 TB Utrecht, The Netherlands; 4 Tranzo, Scientific Centre for Transformation in Care and Welfare, Tilburg University, P.O. Box 90153, 5000 LE Tilburg, The Netherlands

## Abstract

**Background:**

Innovative approaches are needed to support patients' adherence to drug therapy. The Real Time Medication Monitoring (RTMM) system offers *real time *monitoring of patients' medication use combined with short message service (SMS) reminders if patients forget to take their medication. This combination of monitoring and tailored reminders provides opportunities to improve adherence. This article describes the design of an intervention study aimed at evaluating the effect of RTMM on adherence to oral antidiabetics.

**Methods/Design:**

Randomised Controlled Trial (RCT) with two intervention arms and one control arm involving diabetes type 2 patients with suboptimal levels of adherence to oral antidiabetics (less than 80% based on pharmacy refill data). Patients in the first intervention arm use RTMM including SMS reminders and a personal webpage where they can monitor their medication use. Patients in the second intervention arm use RTMM without SMS reminders or webpage access. Patients in the control arm are not exposed to any intervention. Patients are randomly assigned to one of the three arms. The intervention lasts for six months. Pharmacy refill data of all patients are available from 11 months before, until 11 months after the start of the intervention. Primary outcome measure is adherence to oral antidiabetics calculated from: 1) data collected with RTMM, as a percentage of medication taken as prescribed, and as percentage of medication taken within the correct time interval, 2) refill data, taking the number of days for which oral antidiabetics are dispensed during the study period divided by the total number of days of the study period. Differences in adherence between the intervention groups and control group are studied using refill data. Differences in adherence between the two intervention groups are studied using RTMM data.

**Discussion:**

The intervention described in this article consists of providing RTMM to patients with suboptimal adherence levels. This system combines real time monitoring of medication use with SMS reminders if medication is forgotten. If RTMM proves to be effective, it can be considered for use in various patient populations to support patients with their medication use and improve their adherence.

**Trial registration:**

Netherlands Trial Register NTR1882

## Background

Adherence can be defined as the extent to which a person's behaviour - taking medication and/or executing lifestyle changes, corresponds with agreed recommendations from a health care provider [1]. Many patients, especially patients with a chronic illness, experience difficulties in following treatment recommendations. Adherence to long-term therapy for chronic illnesses in developed countries averages only 50% [1]. As a result of poor adherence, patients do not receive optimal benefit from their drug therapy. Suboptimal treatment can lead to increased use of health care services (acute care and hospitalizations), reduction in patient's quality of life, and increased health care costs (drug costs and medical costs) [1-5]. Thus, improving adherence receives world-wide attention. In 2003, the World Health Organization emphasized that "increasing the effectiveness of adherence interventions may have a far greater impact on the health of the population than any improvement in specific medical treatments" [1]. Many interventions to improve patients' adherence to medication have been carried out in the last decades, but most of them did not show significant effects on adherence. Even with the most effective interventions, improvements in adherence and treatment outcomes were generally small [6-9].

Evidently, there is a need for innovative approaches to support patients in following prescribed therapy [6]. Most promising are interventions that are both simple for the patient and relatively easy to implement in daily clinical practice [8,10]. One such innovative approach is the recently developed Real Time Medication Monitoring (RTMM) system. This system is an innovative adaptation of the well-known Medication Event Monitoring System (MEMS). MEMS uses an electronic medication bottle with a microprocessor incorporated into the cap that records the date and time the bottle is opened. MEMS provides an objective and reliable measure of adherence to prescribed medication [11,12] and it has been used to measure medication adherence of various patient populations, including those with asthma [13], diabetes [14], schizophrenia [15] and heart failure [16]. Moreover, MEMS appears to improve adherence slightly, especially when the system is used for a short time [17]. The new RTMM system also uses an electronic medication dispenser which monitors patients' medication use but, as opposed to MEMS, it registers this data in *real time *at a central server. This real time information is directly available through the internet for patients as well as for their care providers. Furthermore, the RTMM system combines monitoring of medication use with a reminder aspect. An explorative study in which patients were reminded daily of their medication intake through a short message service (SMS) showed short-term improvement of adherence among diabetes type 2 patients [18]. However, the effects diminished over time. This might have been the result of the SMS reminders becoming a routine, as in this study they were sent before every intake, and were not triggered by the actual medication use. As the RTMM system registers medication intake data in real time, it is possible to remind patients only when necessary, that is when they actually forget to take their medication. Patients who take their medication in time do not receive SMS-alerts. In this way habituation, and the possible loss of effectiveness associated with it, can be averted. As such, the RTMM system provides opportunities to improve adherence to medication.

Interventions using reminders to improve adherence, such as the one described in this article, are primarily based on the principles of behavioural learning theory, one of the five theoretical perspectives on adherence outlined by Leventhal and Cameron [19]. According to this theory, behaviour depends on stimuli or cues, either internal (thoughts) or external (environmental cues), which elicit certain behaviour. As such the desired behaviour can be learned and maintained by automation after sufficient repetition. The intervention tested in this study aims to modify the behaviour of non-adherent patients by sending external stimuli, in this case SMS reminders.

In addition to the simplicity for the patient and the feasibility of implementation in busy daily practice, interventions also need to be tailored to the patients' reasons for not adhering to medication treatment. Here two types of non-adherence can be distinguished: intentional non-adherence where the patient deliberately misses or alters the doses, and unintentional non-adherence where the patient simply forgets to take the medication. Interventions aimed at improving adherence should address these two types separately [20-22]. Increasing one's awareness of the benefits of the medication is unlikely to improve adherence in patients who unintentionally fail to adhere to medication. Likewise, reminding patients to take their medication will not be helpful for patients who decide to miss or alter their dosage on purpose. Although both types of non-adherence are common, patients more often report unintentional non-adherence [21,22]. RTMM can be especially useful in improving adherence for these patients who forget to take pills or are inaccurate in their timing.

A small pilot project reported patients' positive experiences with this new system [23]. However, the effects of the system on adherence levels are yet to be investigated. This study proposes a Randomised Controlled Trial (RCT) to evaluate the effect of RTMM on medication adherence in patients with type 2 diabetes. The prevalence of diabetes is high and continues to increase. Adherence to oral antidiabetics ranges from 36% to 93% [24]. Improving adherence in diabetes patients leads to better glycaemic control and could in the long-term reduce the incidence of micro- and macrovascular complications associated with diabetes [25,26]. Furthermore, an increase in adherence in diabetes patients can lead to a net reduction in health care costs [3].

The main objective of the intervention study described in this article is to evaluate the effect of RTMM with SMS reminders on the adherence to oral antidiabetic medication of diabetes type 2 patients with suboptimal levels of adherence. The central research question that will be addressed in this study is: To what extent does adherence to oral antidiabetic medication improve in patients who use the RTMM system with SMS reminders compared to 1) patients who use the RTMM system without SMS reminders, and 2) patients who receive usual care?

This article describes the design of this study and discusses the strengths and limitations of the study protocol. The results will be published in separate articles.

## Methods/Design

### Design

**Figure 1:**
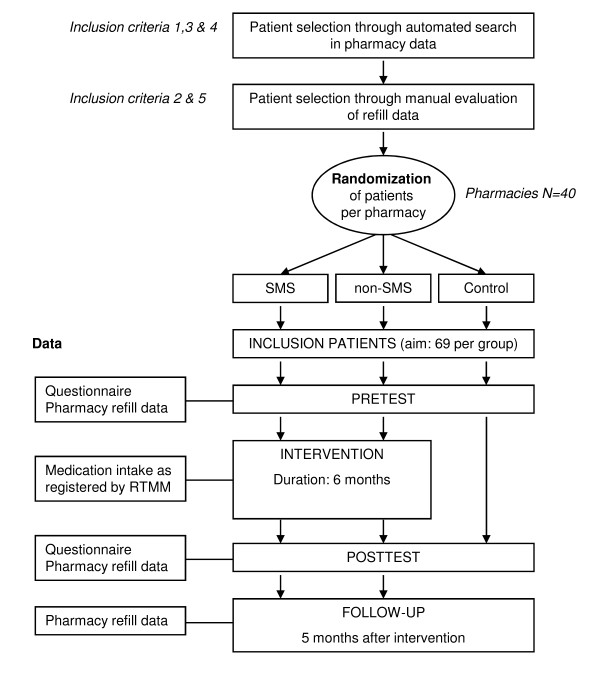
**Flowchart of the intervention study**.

This study is a Randomised Controlled Trial (RCT) with two intervention arms and one control arm involving diabetes type 2 patients selected from 40 pharmacies belonging to Mediq, a Dutch pharmacy chain. Patients are randomly assigned by the research group to one of the three groups. Patients are invited by their pharmacy to participate in the study. Patients in the two intervention groups use the RTMM system either with SMS reminders (first intervention group) or without SMS reminders (second intervention group) for a period of six months. Patients in the control group are not exposed to any intervention. Pre- and post-tests as well as a follow-up test are performed. A flowchart of the study is shown in Figure 1.

### Ethical approval

The Medical Ethics Committee of the Utrecht Academic Medical Centre approved the protocol (METC protocol number 08-165/C). The anonymity of every patient is guaranteed because all data are coded. Neither NIVEL, nor the head office of Mediq, nor Evalan (the supplier of the RTMM system) has access to information on individual patients. Only the pharmacies are able to link patient codes to individual patient information. Furthermore, all participants sign informed consent.

### Participants

The inclusion criteria for participants in this study are:

1) *Using oral antidiabetic medication for at least one year*.

There are three phases in pharmacotherapy: acceptance of the treatment plan, execution of the drug regimen and discontinuation of dosing [27]. We include patients who are beyond acceptance, the first phase, thus patients who have used their medication for at least one year. As such we deliberately exclude patients who have not yet accepted their treatment as these patients might be at increased risk of early discontinuation of the therapy.

2) *If insulin is used in combination with oral medication: using insulin for at least six months*.

Patients who only recently initiated insulin therapy along with their oral antidiabetics, within the past six months, are excluded as these patients might still be experimenting with the right combination of insulin and oral antidiabetics.

3) *Having a refill adherence of less than 80% calculated from pharmacy dispensing data from 11 months preceding the intervention*.

One of the recommendations of Van Dulmen et al. is to focus interventions on patients with suboptimal levels of adherence [8]. The reason to select patients who have an adherence level lower than 80% is that these patients are more likely to benefit from the proposed intervention. The 80% cut-off point is used because missing 20% of the daily prescribed doses is a relevant gap between prescribed and used medication. We estimated that this cut-off point should provide sufficient eligible patients per pharmacy to participate in the intervention. A lower cut-of point would result in more pharmacies having to participate and this would lower the feasibility of the study.

4) *Aged between 18 and 65 years*.

Patients older than 65 years are excluded because they frequently have a more complex clinical picture, with several (chronic) diseases, more complications, and more different medication to take.

5) *Collected the last prescription for oral antidiabetics within the two months prior to the intervention*.

Patients should have collected their prescription recently, that is within two months, to exclude patients who might have stopped taking their medication completely.

6) *Having knowledge of the Dutch language*.

The questionnaires and the written patient information about the RTMM system are in Dutch; therefore patients need to understand the language.

7) *Using a mobile phone*.

The intervention aims at improving adherence through SMS reminders if necessary, thus patients have to be in possession of a mobile phone.

Patients who meet inclusion criteria one, three and four are selected through an automated search in the pharmacy data of 40 pharmacies. Patients who do not meet criteria two and five are excluded on the basis of manual evaluation of the refill data of the selected patients. Patients are randomly assigned, per pharmacy, by the research group to one of the three groups. The age and gender distribution in the three groups as well as the number of patients who use insulin beside oral antidiabetics are evenly distributed in the three groups. This type of randomization is known as restricted randomization. The patient numbers, along with the group assignments, are communicated to the pharmacies. The last two criteria, having knowledge of the Dutch language and possessing a mobile phone, are not available from the pharmacy data and are therefore asked when the patient is invited to the pharmacy.

### Intervention

The intervention consists of providing RTMM with SMS reminders to diabetes type 2 patients who have suboptimal adherence levels to improve their adherence to oral antidiabetic medication. Patients are invited by their pharmacy to participate in the study. Pharmacists and/or pharmacy assistants are instructed by members of the project team about the procedure how to include patients. The instruction covers how to approach patients, provide the RTMM system and inform patients about the intervention.

Patients in both intervention groups receive their oral antidiabetics in the electronic medication dispenser. During the six-month intervention period, the actual medication use of patients is registered in real time at a central database, hosted by the supplier of the RTMM system, Evalan. The main researcher (Vervloet) receives an account with which she can log onto this database at any time to retrieve the data. Patients in the first intervention group, from here on called SMS group, receive a SMS reminder if they do not open their medication dispenser within an agreed time period. These time periods are chosen by the patient during the intake in the pharmacy. The number of specified time periods is equal to the number of daily doses prescribed by the general practitioner. One time period is specified for a once daily dose regimen, two time periods are specified for a twice-daily dose regimen and three time periods are specified for a three times daily dose regimen. For every patient, the pharmacy staff is provided with a form on which these time periods and the patient's mobile number can be completed. This form is sent to Evalan, where the dispenser is programmed accordingly. These time periods are also communicated to the main researcher. The SMS reminder is sent at the end of this period when the dispenser has not been opened. Patients, as well as their pharmacists, receive an internet account through which they can monitor their medication use. Patients in the second intervention group, from here on called non-SMS group, receive their medication in a similar medication dispenser, but without SMS reminders and an internet account. The medication use of these patients is also registered in real time at a central database. Patients in the third group, the control group, receive usual care. They are not exposed to any intervention and they are not approached during the six months.

### Measurements

The medication use of patients in both intervention groups is registered by the RTMM system for a period of six months. Pharmacy data are collected for all participating patients over a total period of 22 months. This comprises 11 months prior to the intervention, for a pre-test, six months during the intervention, for a post-test, and five months after the intervention, for a follow-up test. Data include patient characteristics such as age and gender, and the quantity and regimen information of all dispensed medication.

Primary outcome measure is the adherence to oral antidiabetic medication, which is calculated from data collected through electronic monitoring (RTMM) and pharmacy refill data.

1) Adherence calculated from data registered by the RTMM-system is defined as:

a. the number of medication doses taken divided by the number of prescribed doses;

b. the number of medication doses taken within the correct time interval, the time period being agreed with the patient, with a range of x hours after this period divided by the number of prescribed doses;

These outcome measures are used to study differences in the level of adherence between the two intervention groups during the intervention. In addition, the effect of the SMS reminders on taking medication on time is studied. The proportion of patients who need reminders is studied as well as changes in this proportion over time. Furthermore, by *varying the cut-off points *for adherence we investigate the effects of this variation on the outcome using sensitivity analysis.

2) Adherence as calculated from pharmacy refill data is defined as the number of days for which the oral antidiabetic medication is dispensed during the study period divided by the total number of days of the study period.

This outcome measure is used to study differences in the level of adherence between the three groups before and after the intervention. Moreover, it is used to study whether the adherence of the two intervention groups differs from the control group during the intervention.

Furthermore, two questionnaires, one pre-test and one post-test, are completed by all participants. For comparability, questions at post-test are mostly similar to those at pre-test. Socio-demographic factors such as age, gender, education level and ethnicity, are asked only in the pre-test questionnaire. Both questionnaires contain questions about factors that may be associated with adherence, such as patients' beliefs about their diabetes medication and their illness perceptions. Questions about patients' experiences with RTMM are added to the post-test questionnaire for patients in both intervention groups.

### Sample size calculation

Sample size calculations are based on the expected effect of the intervention on the primary outcome which is the adherence. However, as this is the first study on the effects of RTMM on levels of adherence, there are no data on which to base our expectations. Most studies on improving medication adherence have relatively small effects. We, therefore, hypothesize that the difference in adherence level between the SMS group and control group at the end of the intervention is 10 percent. Power analysis showed that, using a one-sided t-test (alpha = 0.05) for a 10 percent difference (SD = 20), with a power of 0.90, 69 patients are needed in each arm.

### Data analysis

Data are analysed based upon an intention-to-treat principle [28]. All patients, regardless of whether they actually finish the intervention, are included in the analysis. Descriptive analyses are obtained about the demographic characteristics of patients and other control variables in both the pre- and post-test in all three groups. Differences are tested using chi-square or t-tests. To test the effect of the intervention a multilevel design is used, since the data have a nested structure. Medication events are clustered within patients, and patients are clustered within pharmacies. The primary outcome, the adherence, is the dependent variable. All independent variables of importance, for example socio-demographic factors and medication regimen, are included in the model to adjust for these variables. Data are analysed with Stata 10.0 for Windows and MLwiN 2.11 for multilevel modelling.

## Discussion

The intervention study described in this article is based on the innovative Real Time Medication Monitoring (RTMM) system. This system is the first to combine real time monitoring patients' medication use with sending reminders through SMS only when medication is actually forgotten. Reminding per SMS is a relatively simple method with low intrusiveness [29]. As such, RTMM appears to be a simple intervention for patients. Haynes and colleagues concluded in their review that for short-term treatments, quite simple interventions increased adherence. For long-term treatments, only some complex interventions led to improvements in health outcomes [9]. However, complex interventions are difficult to implement, time-consuming and very labour-intensive. Simple interventions that are relatively easy to implement in busy daily practice are most promising [8,10]. This conclusion was shared by a forum of twenty internationally renowned experts in the field of adherence [30]. Furthermore, the intervention is aimed at patients with suboptimal levels of adherence. These are the patients who could benefit most from interventions aimed at improving adherence. However, the identification of these patients appears to be difficult [10]. We believe that through our selection criteria we will capture a group of non-adherent diabetes type 2 patients. However, it has yet to be proved that our criteria have indeed selected a group of patients for whom an intervention such as RTMM can be beneficial.

### Strengths and limitations

A common critique of electronic medication monitoring based on the time and date the medication dispenser is opened, is that it cannot be confirmed that the medication is actually taken or that no more or no less than the prescribed dose is taken. Only drug assays can confirm ingestion. However, studies comparing the sequence of medication events with projected and periodically measured concentrations of the drug in plasma, confirmed the validity of medication event monitors. Mismatches between medication events and actual dosing were too rare to create substantial differences between projected and actual concentrations of the drug in plasma [31-34]. Moreover, other methods to measure adherence such as self-report and pill count tend to overestimate adherence [35].

In addition, there is no consensual standard of 'good adherence', because the level of adherence necessary to achieve the desired effect varies between medications and between and within patients [36]. We used the cut-off point of 80% to label patients as adherent (above 80%) or non-adherent (below 80%). Although arbitrary, this cut-off point is more commonly used in studies on adherence [2,37,38]. Furthermore, a lower cut-off point would result in smaller numbers of patients per pharmacy eligible to participate in the intervention. More pharmacies would then be needed to achieve desired patient numbers, lowering the feasibility of the study.

Participation is restricted to pharmacies belonging to one pharmacy chain. The success or failure of the intervention could therefore be attributed to specific characteristics of pharmacies within this chain. However, the diversity of pharmacies within this chain is large and a Dutch study has shown that being part of a chain does not make a difference in the quality of care as perceived by patients [39].

Theoretically, it is possible for the supplier of RTMM (Evalan) to manipulate the data on medication use registered at the central database. However, both patients and pharmacists have direct access to the data, they can log onto this database at any given time to view the patients' medication use. Furthermore, the primary outcome of this study which is adherence to oral antidiabetics, is not only based on RTMM data, but also on pharmacy refill data. Manipulation of the RTMM data would result in a discrepancy between the RTMM data and the refill data (internal inconsistency). Most importantly, the ultimate effect of the intervention is determined by analyzing pharmacy refill data, since for the control group no RTMM data is available.

The management of diabetes involves a healthy lifestyle and long-term adherence to treatment, aimed at preventing or delaying microvascular and macrovascular complications. The main health outcome in diabetes type 2 patients is to achieve and maintain good glycaemic control, that is an optimal blood glucose level (HbA_1c_). One of the factors that contribute to achieving glycaemic control is therapy with antidiabetic medication. Therefore, medication adherence is an intermediate outcome measure. However, several studies have shown that improvement of medication adherence leads to better glycaemic control [26,40,41].

## Conclusion

If this intervention, based on providing RTMM with SMS reminders to diabetes type 2 patients with suboptimal adherence to achieve higher adherence levels, proves to be effective it can be considered for use in various patient populations to support patients in their medication use and improve their adherence.

## Competing interests

The authors declare that they have no competing interests.

## Authors' contributions

MV and LvD drafted the manuscript. LvD and JS designed the study. MV, LvD, JS and BvV were involved in the implementation of the study. JS, MB and DdB critically revised this manuscript. All authors read and approved the final version of the manuscript.

## Pre-publication history

The pre-publication history for this paper can be accessed here:

http://www.biomedcentral.com/1472-6963/11/5/prepub
